# Finding Excited-State Minimum Energy Crossing Points on a Budget: Non-Self-Consistent Tight-Binding Methods

**DOI:** 10.1021/acs.jpclett.3c00494

**Published:** 2023-05-05

**Authors:** Philipp Pracht, Christoph Bannwarth

**Affiliations:** † Yusuf Hamied Department of Chemistry, University of Cambridge, Lensfield Road, Cambridge CB2 1EW, United Kingdom; ‡ Institute for Physical Chemistry, RWTH Aachen University, Melatener Str. 20, 52074 Aachen, Germany

## Abstract

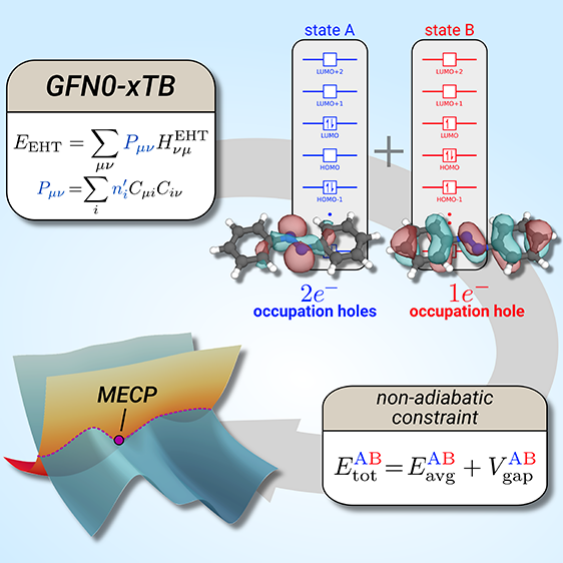

The automated exploration and identification of minimum energy conical intersections (MECIs) is a valuable computational strategy for the study of photochemical processes. Due to the immense computational effort involved in calculating non-adiabatic derivative coupling vectors, simplifications have been introduced focusing instead on minimum energy crossing points (MECPs), where promising attempts were made with semiempirical quantum mechanical methods. A simplified treatment for describing crossing points between almost arbitrary diabatic states based on a non-self-consistent extended tight-binding method, GFN0-xTB, is presented. Involving only a single diagonalization of the Hamiltonian, the method can provide energies and gradients for multiple electronic states, which can be used in a derivative coupling-vector-free scheme to calculate MECPs. By comparison with high-lying MECIs of benchmark systems, it is demonstrated that the identified geometries are good starting points for further MECI refinement with *ab initio* methods.

Photochemical processes have become an increasingly important way to steer molecular reactions and motions^1,2^ and are routinely aided by computational studies. Describing the associated photoreactions through computational chemistry requires the treatment of non-adiabatic couplings to describe non-radiative transitions between the different electronic states.^3,4^ Representative geometries for the corresponding non-adiabatic transitions are those structures of minimum energy that are located along the seam between energetically close potential energy surfaces (PES) of the electronic states, i.e., the minimum energy crossing point (MECP) and minimum energy conical intersection (MECI) geometries. These geometries play a crucial role in transitions between different electronic states and fulfill a similar purpose for photochemical processes as transition state geometries in a ground state reaction.^5,6^

In spin-allowed photoreactions, many local MECIs are often accessible from the so-called Franck–Condon (FC) region. Identifying these MECI geometries, or at least plausible candidates, is essential for the computational modeling of excited-state photoreactions. The plausible candidates are MECPs between diabatic states, which are typically close to a true MECI between adiabatic states.^7^ Since no derivative coupling vectors are required for describing the MECP, the computational complexity is greatly reduced compared to MECI optimizations. Approaches to explore the associated MECI space by locating MECPs have been presented by the groups of Mitrić^8^ and Martínez.^4^ We recently presented a metadynamics-based MECP screening workflow leveraging the efficient semiempirical extended tight-binding methods (xTB).^9,10^ A surprisingly good performance was observed for finding MECPs in combination with the GFN2-xTB method.^11,12^ Using a gap-dependent potential, the MECPs between the lowest low-spin and high-spin potential energy surfaces were identified and shown to closely resemble *S*_1_/*S*_0_ MECIs obtained at a higher level of theory. However, a limitation is the use of a self-consistent field (SCF) treatment that can, in principle, only produce the ground state within a given spin multiplicity. Consequently, previous studies were limited to identifying MECPs resembling the *S*_1_/*S*_0_ MECIs or the *T*_1_/*S*_0_ MECPs. However, crossings between higher-lying electronic states are essential in many photochemical processes. Typical examples are *T*_2_/*T*_1_ and *S*_2_/*S*_1_ conical intersections^4,6,13,14^ as well as *T*_1_/*S*_1_ MECPs, which play an important role in the so-called thermally activated delayed fluorescence.^15^ Identifying such points is impossible with the previously described approach,^9^ since the SCF solutions will collapse back to the respective ground state.

In this work, we therefore discuss an extension of the previous scheme that allows the quick calculation of higher-lying states and their associated MECPs, made possible by employing a non-self-consistent xTB treatment.^12,16^ The proposed method is characterized by requiring only a single diagonalization of the Hamiltonian to determine the necessary orbitals. The desired states are then approximated by a single determinant generated by different occupation vectors that also provide access to non-Aufbau occupations. A modified version of Fermi smearing allows smearing of electrons and occupational holes, which provides robustness during optimization and dynamics simulations. Herein, the non-self-consistency provides a typical speed-up of 1–2 orders of magnitude compared to the already inexpensive GFN2-xTB method while providing nearly identical MECP geometries.

As in previous work, we are pursuing a “derivative coupling vector-free” treatment^9,17−21^ that provides MECPs of two (or more) diabatic multidimensional PESs. These points can then serve as approximations to MECIs between adiabatic states that are obtained from multideterminant wave functions.^7^ The idea here is to locate the MECP on an artificial seam PES given by
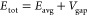
1combining two core components *E*_avg_ and a penalty function *V*_gap_. For the considered *n* electronic states of the molecule, the seam PES is based on the arithmetic mean
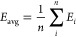
2This energy average is combined with a pairwise penalty
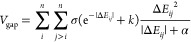
3minimizing the energy gap |Δ*E*_*ij*_| between two diabatic states *i* and *j*. The latter potential confines the overall energy to a lower dimensional hyperline where the states are degenerate. Inclusion of *E*_avg_ then helps to locate the minima on this hyperline that correspond to the MECPs.^5^ A key feature of this procedure, and the penalty functions in particular, is their ability to locate transition state analogues, i.e., the MECP, involving discontinuous first and second derivatives in the adiabatic framework.^22^ In eq 3, a total of three empirical parameters, σ, *k*, and α, are chosen to converge the energy gap to zero near the seam region while providing a linearly growing energy bias far away from it.^9,18^ Detailed adjustment of the empirical parameters in the penalty potential may be required for some systems, but manually selected values of σ = 10.0, α = 0.005, and *k* = 0.25 often work well and were used throughout this study. While the σ and α parameters were chosen in accordance with earlier studies,^9,18^*k* was selected to provide the linearly growing bias far away from the potential minimum. Selecting too small values for *k* will result in the formation of artificial maxima around the MECP. System dependent adjustment of *V*_gap_ should be done mainly through the interaction strength σ. The combination involving eq 1 is robust and may generally be extended, e.g., by metadynamics-based sampling techniques^10,23,24^ or global optimization procedures,^25,26^ to explore a larger portion of the crossing seam hyperplane.

One limiting factor for the MECP calculation is the ability of theoretical models to simultaneously describe the different electronic states near the intersection. While typically associated with costly high level *ab initio* calculations,^3,27^ application of much less costly semiempirical approaches has proved to perform well.^28−31^ Within the derivative coupling vector-free treatment, large reduction of the computational cost is achieved in combination with semiempirical methods of the GFN*n*-xTB level,^11,12,32^ which provide reasonable estimates of *S*_0_/*S*_1_ MECIs via the *S*_0_/*T*_1_ MECP.^9,33^ However, as discussed previously, only lowest-energy solutions for *a priori* specified differences in the net α and β occupation can be enforced in this framework. In consequence, states that involve higher energetic electronic states, or simply *holes* in the net occupations, are inaccessible within the GFN*n*-xTB framework. To remedy this problem, we make use of a less well-known non-self-consistent variant of GFN*n*-xTB methods, entitled GFN0-xTB.^12,16^ The energy in GFN0-xTB is defined by
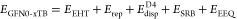
4where *E*_rep_, *E*_disp_^D4^, *E*_SRB_, and *E*_EEQ_ are classical repulsion, dispersion, short-range, and charge equilibrium electrostatics terms, respectively.^12,16^ The electronic energy within this method is given solely by the extended Hückel-type (EHT) energy
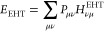
5where *P*_*μν*_ is the valence electron density matrix in the non-orthogonal atomic orbital (AO) basis and *H*_*νμ*_^EHT^ is the extended Hückel theory (EHT) type matrix. It is the single-particle treatment, reflected in eq 5, that enables an approximate treatment of higher-lying electronic states in the proposed procedure: For a given set of atomic coordinates, the Roothaan–Hall-type equations^34,35^**H**^EHT^**C** = **SCϵ** only need to be solved once, due to the purely one-electron “extended Hückel-type” Hamiltonian. Hence, the Hamiltonian matrix is independent of the electron distribution and, using other occupations differing from the Aufbau occupation, gives direct access to excited states in the one-electron approximation. Consequently, one only needs a single matrix diagonalization to calculate GFN0-xTB energies for multiple diabatic states from *P*_*μν*_ = ∑_*i*_ *n*_*i*_^′^*C*_*μi*_*C*_*iν*_ by modifying the orbital occupation *n*_*i*_^′^ = *n*_*iα*_^′^ + *n*_*iβ*_^′^ accordingly (see below) and employing it in eq 5. This treatment drastically differs from the SCF procedure in other xTB variants^11,32^ and enables the computation of energies and gradients with arbitrary orbital occupation. Furthermore, since the diagonalization of the *n* × *n* Hamiltonian matrix, with formal scaling of , is the cost-determining step of the xTB calculation, significant savings of computation time are achieved by replacing the SCF with a non-self-consistent treatment. For the determination of MECPs, the use of GFN0-xTB will accelerate calculations by an additional factor since the multiple states can be obtained from the same diagonalization of the EHT matrix by simply changing the occupation.

As mentioned earlier, an important aspect is the treatment of spin multiplicities at our chosen level of theory. All GFN*n*-xTB variants have no spin-discriminating terms in the Hamiltonian and, furthermore, use a spin-restricted formalism.^12,32^ Hence, the respective α and β orbitals have identical orbital energies and spatial parts and it is impossible to distinguish open-shell states with identical orbital occupation but different multiplicity. To mimic the simulation of multideterminant states via a single-determinant, orbitals may have non-integer, i.e., fractional occupation numbers (FON). We use Fermi smearing which allows us to handle near degeneracies.^11,36^ For our current application, a modified FON *n*_*iσ*_^′^ for the *i*th spin-molecular orbital ψ_*iσ*_ (σ = {α, β}) is given by
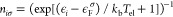
6
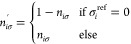
7where *k*_b_ is Boltzmann’s constant and ϵ_*i*_ is the orbital energy of ψ_*i*_. The key novelty here is that the FON allows smearing of configurations that have holes in the net reference occupation σ_*i*_^ref^. These holes can be added separately to the occupation vector of α or β spins to provide non-Aufbau occupations. The modified Fermi smearing in eq 7 allows higher energetic MOs (placed above the predefined holes) to partially smear “back” into the lower-lying hole orbitals. This formulation enhances the numerical stability of the overall workflow during optimization and molecular dynamics runs. As usual, the Fermi level ϵ_F_^σ^ in the respective α or β orbital space is initially obtained as the average  and then adjusted to match the total number of α and β electrons. The electronic temperature *T*_el_ serves as a simple adjustment parameter to steer the amount of smeared occupations and can often be set to a rather high value. Note that no electronic entropy term −*T*_el_*S*_el_ is calculated from the FON for this specialized application of GFN0-xTB, which in contrast to regular xTB schemes ensures that eq 3 approaches zero close to the MECP.^9,12^ In summary, the procedure employs GFN0-xTB (eq 4) to calculate energies and gradients for multiple diabatic states. The latter are obtained from a single diagonalization of the Hamiltonian matrix using different orbital occupations. If employed in molecular geometry optimization via eq 1, one obtains the corresponding MECPs. As indicated by the GFN abbreviation, which stands for geometries, frequencies, and non-covalent interactions, the goal here is not an exact description of the electronic structure but rather the qualitatively correct identification of low energy molecular geometries at the crossing seam. GFN0-xTB can therefore serve as a low level in multilevel schemes, enabling rapid testing of different net orbital occupations or for providing reference points for further refinement.

To illustrate the capabilities of the introduced scheme, azobenzene provides a helpful benchmarking example. From theoretical studies, mainly, the MECIs and MECPs of azobenzene are known to be dependent on the CNNC dihedral twist of the molecule, and the *S*_0_/*T*_1_ MECP and *S*_0_/*S*_1_ MECI, in particular, are located at around 90°.^37−39^ Performing a scan along the dihedral coordinate reveals the existence of such points at the semiempirical GFN0-xTB level, as shown in Figure 1.

**Figure 1:**
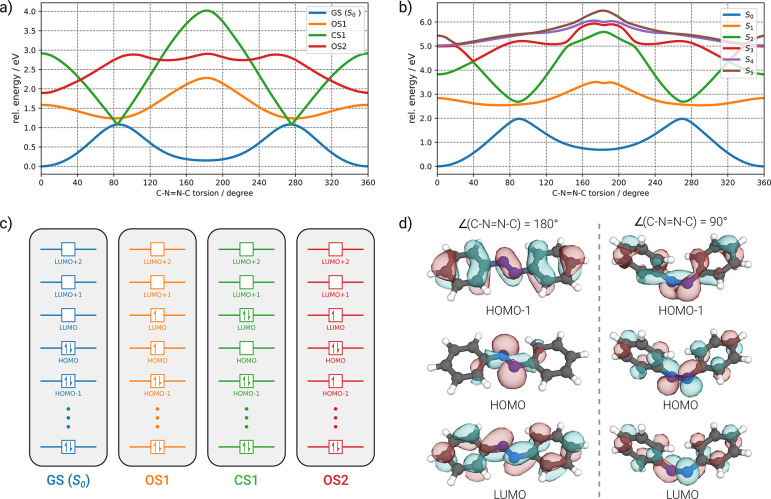
(a) Scan along the C—N=N—C torsional coordinate of azobenzene at the GFN0-xTB level (*T*_el_ = 3000 K). The scan was conducted in 1° steps, and each point was relaxed to the ground state (GS) *S*_0_ geometry while keeping the dihedral angle constrained. Also shown are the two lowest open-shell (OS) energies and the doubly excited closed-shell (CS) energies. (b) Reference single-point energies at the *hh*TDA-BHLYP-D3(BJ)/def2-SV(P) level. The plotted energies (in eV) for different electronic states correspond to a calculation at the given GFN0-xTB *S*_0_ structure. (c) The employed reference occupations for the GFN0-xTB calculation. Note that a clear state classification is not possible due to the spin-restricted treatment (see text). (d) Canonical HOMO–1, HOMO, and LUMO orbitals of azobenzene at C—N=N—C angles of 90° and 180°.

The four different electronic states shown in Figure 1a correspond to the closed-shell ground state (GS) configuration, the lowest open-shell OS1 configuration, and two different configurations that could represent a possible *S*_2_ state: one corresponds to a HOMO to LUMO (*n*^2^π^*2^) double excitation, while the other is a HOMO–1 to LUMO (ππ^*^) open-shell configuration. The latter could either resemble a *S*_2_ or *T*_2_ state, since GFN0-xTB has no spin-discriminating terms in the Hamiltonian.^12^ The OS1 and GS configurations are expected to closely resemble the *S*_1_ and *S*_0_ states near the GS minimum. Any MECP of interest between these configurations can nicely be identified from the scan, in particular a potential tristate *S*_0_/*S*_1_ (or *T*_1_)/*S*_2_ intersection at a CNNC torsion of about 90° (and, symmetrically, 270°). Apart from an overall underestimation of relative energies at the semiempirical level, the reference calculation at the *hh*TDA-BHLYP-D3(BJ)/def2-SV(P) level^38,40^ in Figure 1b reveals a good correspondence of the adiabatic states to the configurations obtained via GFN0-xTB.

As the energies were obtained as single points at the GFN0-xTB geometries, there are no MECIs or proper state crossings observed for the adiabatic states from *hh*TDA. Potential MECI candidates at the *hh*TDA level are, however, clearly visible: the *S*_0_/*S*_1_ intersection at CNNC 90° as well as intersections of the ππ^*^ and *n*^2^π^*2^ states at CNNC dihedral angles of approximately 40° and 140°. The modified GFN0-xTB scheme is clearly capable of qualitatively capturing the overall characteristics of the non-adiabatic potential energy surfaces for azobenzene, which strongly indicates its fitness for screening applications. The latter point is further strengthened by the overall computational cost: Each of the 360 single-point calculations at the *hh*TDA level in Figure 1b had an average wall-time of approximately 25 s utilizing the GPU-accelerated TeraChem program package^41,42^ using 8 cores of an Intel Core i7-11700 (2.50 GHz) CPU together with a NVIDIA GeForce RTX 3070 GPU. In contrast, the *entire* relaxed scan at the GFN0-xTB level takes about 8 s on a laptop using 4 cores of a 2.3 GHz Intel Core i7-11800H CPU.

The data presented in Figure 1a is, to the best of our knowledge, the very first calculation of any extended tight-binding method for higher electronic states involving occupational holes. We test the applicability to describe different electronic states in the proposed one-electron approach using *S*_0_/*S*_1_ intersections from the literature. We refer here to two benchmarks: a set of benzene MECIs calculated at the FOMO-CASCI(6,5)/6-31G level^4,43−46^ and a set of small organic molecule MECIs^28^ calculated at the *hh*TDA-BHLYP-D3(BJ)/def2-SV(P) level.^9,38,40^ While the latter was put forward as a general test set for typical conical intersection geometries, benzene offers an archetypal example of a small molecule with a multitude of MECIs. Comparisons between the GFN0-xTB optimized MECPs and the reference MECIs are shown in Figure 2 where, as a simple but insightful metric for comparison, the root-mean-square deviation (RMSD) of Cartesian coordinates^47^ is given for each of the benchmark cases.

**Figure 2:**
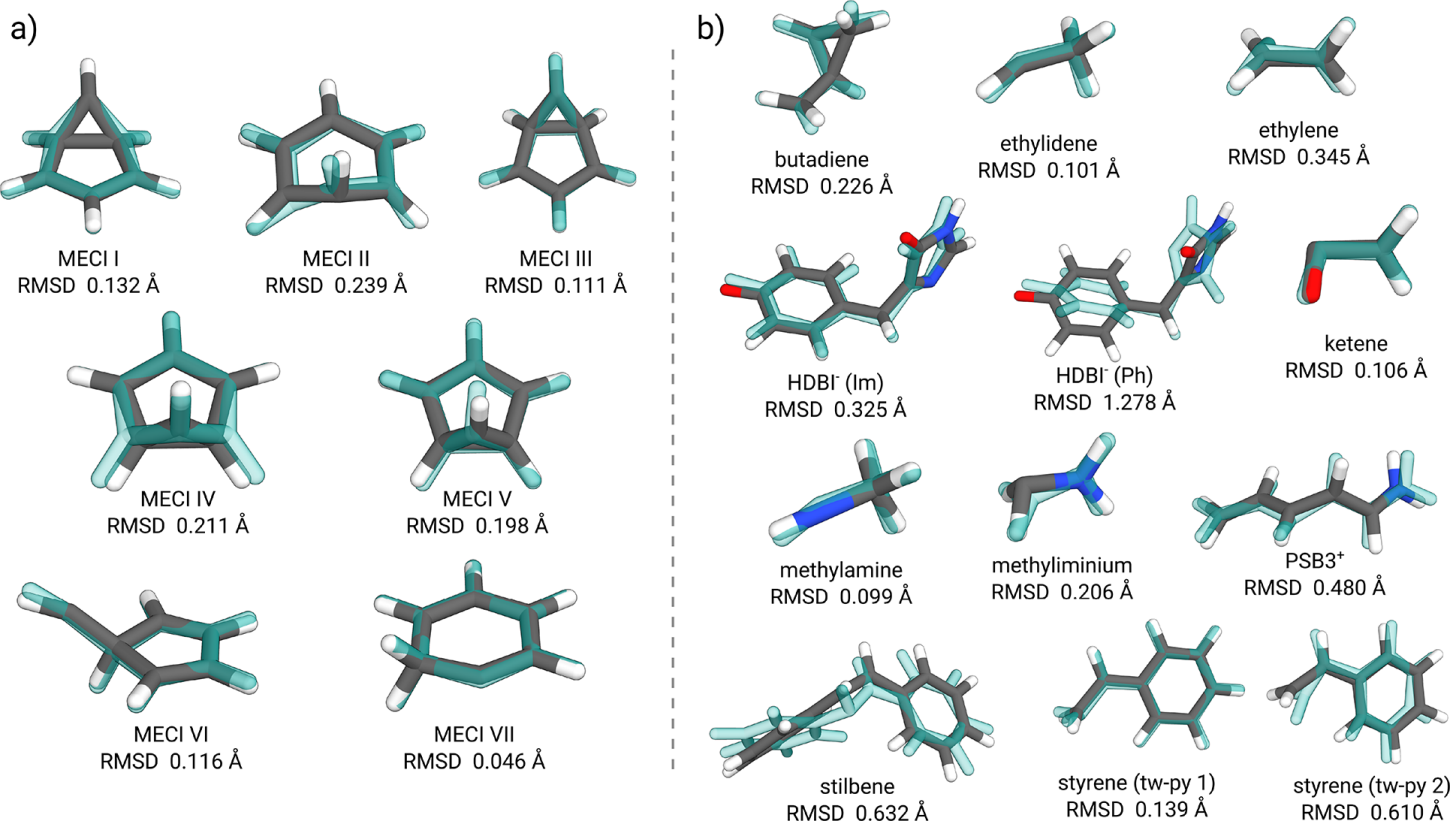
Overlay of GS/OS1 crossing point geometries calculated at the semiempirical GFN0-xTB level (solid color) and (a) reference FOMO-CASCI(6,5)/6-31G *S*_0_/*S*_1_ MECI geometries for the benzene molecule (transparent blue) taken from ref (4) or (b) reference *hh*TDA-BHLYP-D3(BJ)/def2-SV(P) *S*_0_/*S*_1_ MECI geometries for a set of organic molecules (transparent blue) from refs (9) and (28). Structural Cartesian RMSD values are provided below each structure.

An in-depth discussion for these systems can be found in previous work^9^ and the citing literature.^4,28,48^ To summarize the current findings, GFN0-xTB shows virtually identical characteristics as the related self-consistent GFN2-xTB method in the description of GS/OS1 MECPs, which clearly resemble the *S*_0_/*S*_1_ MECI between adiabatic states. RMSDs at the GFN0-xTB level compared to the reference structures are marginally worse than those with GFN2-xTB, as would be expected for this more approximate non-self-consistent method. As observed before, MECP structures at the GFN*n*-xTB level often lack pyramidalization features for non-polar systems compared to the reference MECIs, due to which some details cannot be distinguished, for example, the two styrene CIs.^9^ This finding can mostly be attributed to missing (Fock) exchange in the semiempirical potential.^48^ The “worst case” molecule in the test set, stilbene, suffers from the same missing pyramidalization feature and additionally differs in the torsional conformation of the phenyl groups, which yields a high RMSD despite the qualitative similarity between the MECP and the reference. Nevertheless, the agreement between GFN0-xTB and the reference geometries is overall quite impressive, particularly considering the extremely low computational cost of the method.

Moving on to higher-state MECPs, novel capabilities of the approach are demonstrated. As a first showcase, we refer to a representative “real life” example from a series of joint experimental and theoretical studies of the photochemical deracemization of primary allene amides.^6,33,49^ In a recently studied deracemization photoreaction, two diastereomeric complexes of the chiral allene and chiral photocatalyst are formed, **7**·**8**a and **7**·*ent*-**8**a. It was shown that the distinguishing factor in this process is the *T*_1_/*T*_2_ MECI, where a triplet state is transferred from the catalyst to the allene. The theoretical description of this system is challenging due to its size and the triplet states being located on different sites. Here, we model the *T*_1_ as HOMO–LUMO excitation (cf. OS1 in Figure 1) and the *T*_2_ state as a HOMO–1 to LUMO+1 excitation at the GFN0-xTB level. The resulting geometries are depicted in Figure 3a in comparison with the FOMO-CASCI(4,4)-D3(BJ)/def2-SV(P) reference from ref (6).

**Figure 3:**
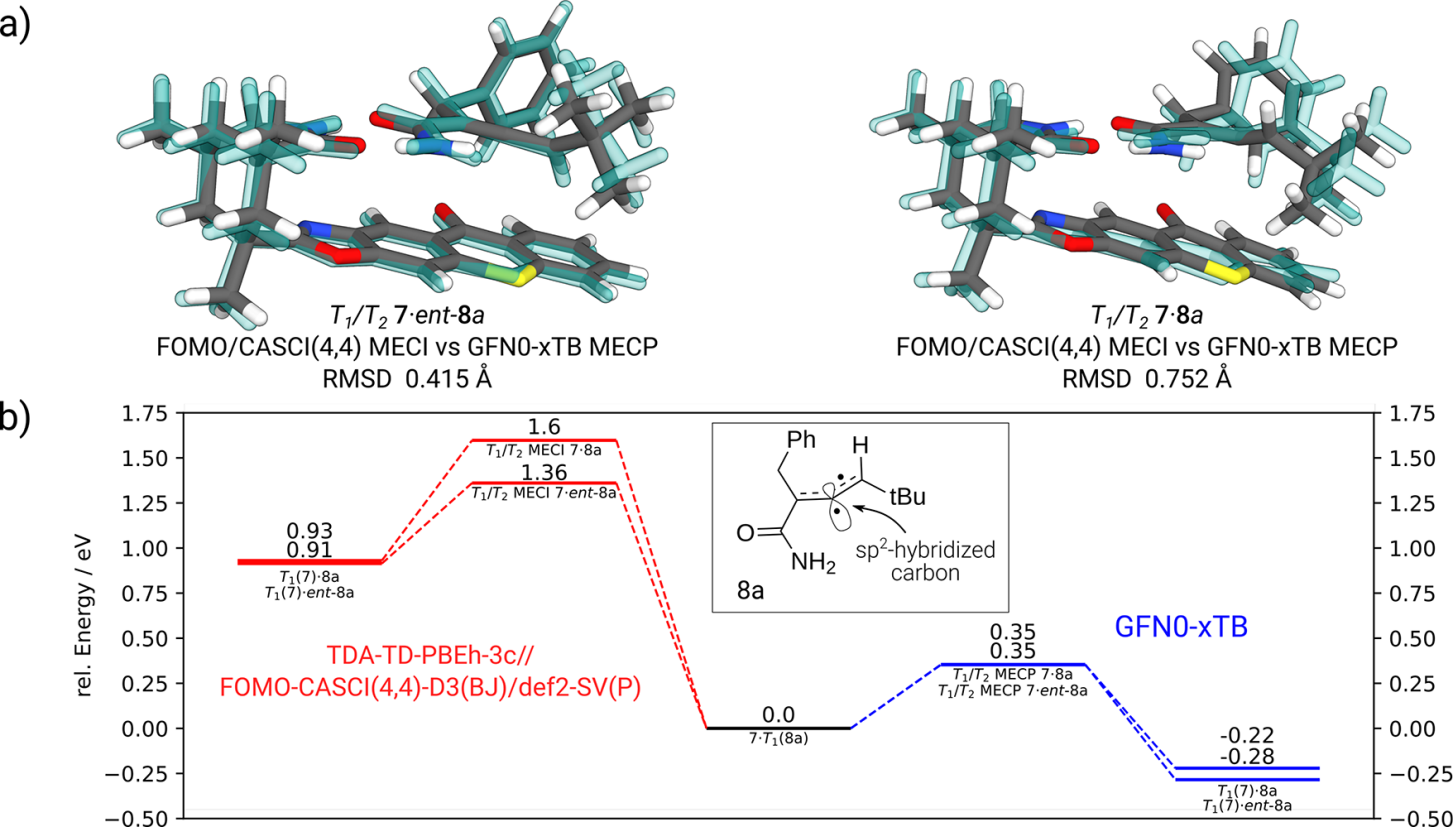
(a) *T*_1_/*T*_2_ MECIs of the **7**·**8**a enantiomers from ref (6) (transparent blue) optimized as MECPs (see text) at the GFN0-xTB level (solid color). (b) Relative energies (in eV) for the Dexter-type energy transfer associated with (a). Energies at the TDA-TD-PBEh-3c//FOMO-CASCI(4,4)-D3(BJ)/def2-SV(P) level (red) are compared with GFN0-xTB energies (blue). At the reference point, the triplet state is located at the then achiral allene **8**a.

Structurally, the triplet state located on the allene, **7**·*T*_1_(**8**a), is characterized by an sp^2^-hybridized central allylic carbon atom (cf. Figure 3b). This achiral form of the molecule is the crucial step during the deracemization, as it is formed primarily from one of the enantiomers. Employing GFN0-xTB, the structural characteristics of the triplet minima and, via eq 1, also the MECPs (as approximations to the MECIs) are reproduced. The relative energies between the structures with a triplet located at the catalyst *T*_1_(**7**)·(ent-)**8**a and the respective MECPs are of similar magnitude (around 0.6 eV) with both the reference method TDA-TD-PBEh-3c//FOMO-CASCI(4,4)-D3(BJ)/def2-SV(P) (shown in red in Figure 3b), and GFN0-xTB (shown in blue). However, the energetic ordering of the substrate complex (triplet located on the catalyst) and of the product complex (triplet located on the then achiral allene) is inverted compared to the *ab initio* treatment. This effect is probably due to the purpose-specific parametrization and the lack of proper electrostatics in the GFN0-xTB method in particular.^12^ While the quantitative calculation of photoreaction pathways is not the goal here, GFN0-xTB provides a sufficiently reasonable, fast, and robust level of theory for the efficient prescreening of plausible MECP structures between higher-lying states, which can then be refined at a higher level of theory.

We further demonstrate the applicability of the GFN0-xTB-based approach to identifying high-lying MECPs for several other molecules in Figure 4. The depicted structures refer to molecules with *S*_2_/*S*_1_ conical intersections that are taken from the literature^4,50−57^ to provide a set of appropriate benchmark examples. Comparison between the GFN0-xTB optimized MECPs and the reference MECIs reveals excellent agreement for the azulene, adenine, and cytosine molecules. Somewhat larger deviations are obtained for the cases of hexatriene, merocyanine, pyrazine, benzene, and fulvene, which differ with regard to one of their defining dihedral or pyramidalization angles, leading to a rather large RMSD, but overall still show the correct structural motif and correct bonding pattern. These differences are quite sensitive to the employed method and can also disagree between the two reference methods. For example, the fulvene MECI converges toward the benzene MECI structure at the *hh*TDA-BHLYP-D3(BJ)/def2-SV(P) level, apparently due to some preference for the doubly excited state. Likewise, the performance of FOMO-CASCI depends strongly on the chosen active space and the electronic temperature used in the orbital generation. For example, hexatriene optimizes to a flat structure at the FOMO(*∞*)-CASCI(4,3)-D3(BJ)/def2-SV(P) level. GFN0-xTB can describe both properly if the corresponding orbital occupation (OS1/OS2 vs OS1/CS1) is used. In Se-guanine, the selenium atom is removed from planarity of the guanine moiety at the reference level, while GFN0-xTB predicts it to be virtually identical to the ground state structure. However, both types of structures exist as *S*_2_/*S*_1_ CIs in the literature.^57^ The largest differences occur for thioanisole, demonstrating the ambiguity in the theoretical description. Here, the CI is characterized by an out-of-plane bend of one of the phenyl carbon atoms. While at the *hh*TDA reference level the latter is at the position of the thiomethyl group, it is the β-carbon for the GFN0-xTB and FOMO-CASCI levels. Both results contradict findings in the literature, where the *S*_2_/*S*_1_ CI is identified as the pathway for a methyl radical dissociation with a corresponding S–C bond elongation and an overall planar geometry.^52^ On the GFN0-xTB side, the approximate monopole-based and non-self-consistent treatment of electrostatics may be responsible for observed deviations, particularly if orbitals other than π orbitals are involved in the respective states. Nonetheless, the examples shown in Figure 4 make a strong case for the proposed method and clearly show that a sensible screening of higher-lying MECPs is possible at a semiempirical xTB level. Computational wall times at the reference levels ranged from 6 min to 1 h 20 min, while at the GFN0-xTB level the optimizations, starting from the GS geometry, take 1 to 2 s, on the same machines mentioned above.

**Figure 4:**
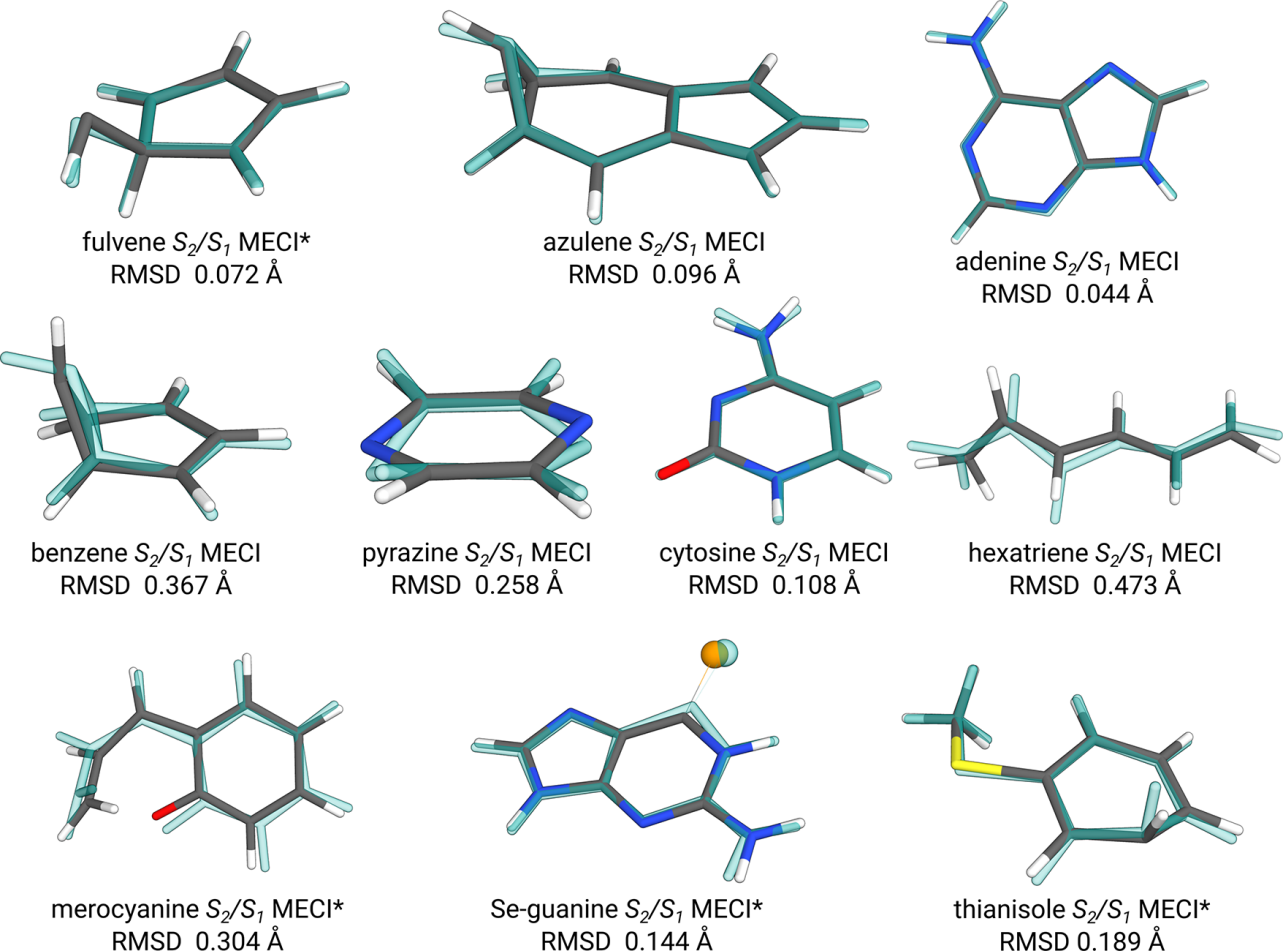
Selection of molecules optimized as the *S*_2_/*S*_1_ MECP at the GFN0-xTB level (solid color) and either FOMO(*∞*)-CASCI(4,3)-D3(BJ)/def2-SV(P) (marked by *) or *hh*TDA-BHLYP-D3(BJ)/def2-SV(P) MECI (transparent blue). The Cartesian RMSD is indicated below each structure. Systems were calculated as OS2/OS1 MECPs at the GFN0-xTB level, with the exception of azulene, benzene, and merocyanine, which are obtained as CS1/OS1 MECPs.

Finally, eq 2 shows a summation of pairwise constraints between two states. The procedure can therefore be used to find MECPs that are close estimates to tristate conical intersections. In fact, tristate CIs are inherently difficult to obtain by conventional optimization procedures, so the derivative coupling vector-free treatment offers an excellent alternative, as recently demonstrated by Baek et al.^21^ Tristate CIs for a number of molecules have been described,^58−60^ but for brevity, we will focus only on the uracil nucleobase.^61,62^ A comparison between the *S*_0_/*S*_1_/*S*_2_ tristate CI calculated at the MRCI(12,9)/cc-pVDZ level and the corresponding GFN0-xTB calculation is shown in Figure 5.

**Figure 5:**
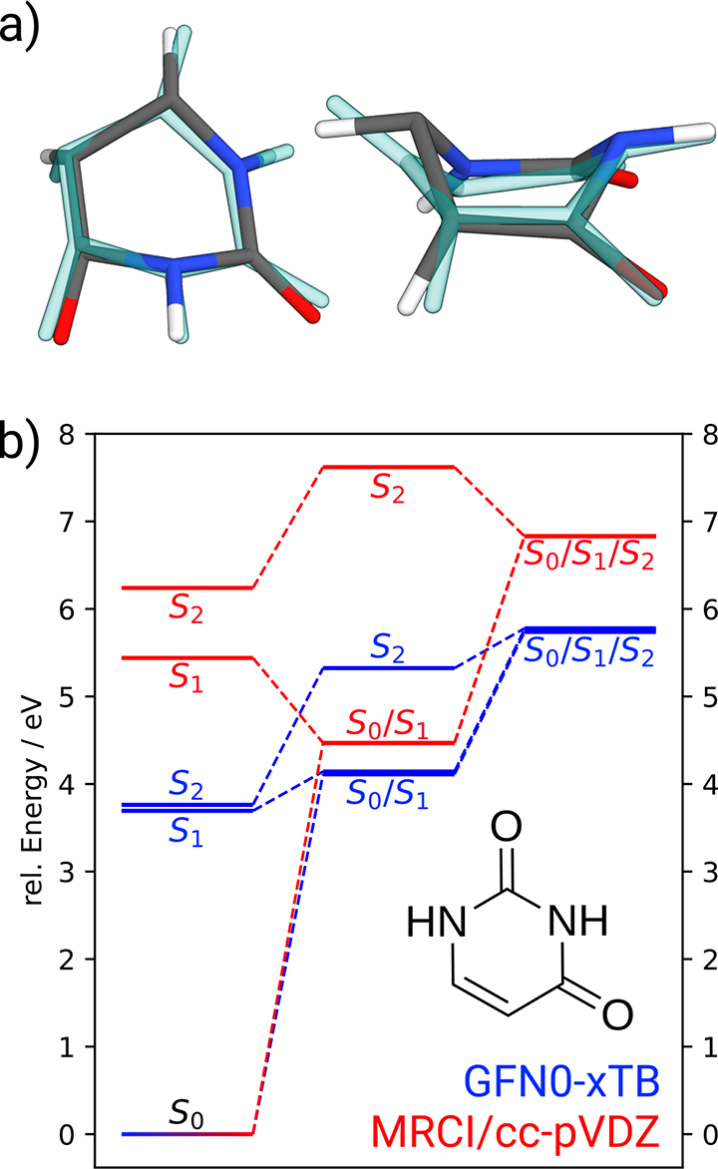
(a) Top- and side-view of the *S*_0_/*S*_1_/*S*_2_ tristate MECP of the uracil nucleobase. Compared are the GFN0-xTB (solid color) and MRCI/cc-pVDZ (transparent blue) geometries. The Cartesian RMSD between the two is 0.263 Å. (b) *S*_0_, *S*_1_, and *S*_2_ energy levels for the Franck–Condon point, the *S*_0_/*S*_1_ MECP, and the tristate MECP. The MRCI(12,9)/cc-pVDZ geometry and energies were reproduced from ref (62).

Despite much less theoretical sophistication compared to the MRCI level used in ref (62), GFN0-xTB is able to generate the tristate MECP closely resembling the given reference. Characteristic structural features that distinguish the tristate from the Franck–Condon point or the *S*_0_/*S*_1_ MECP, especially the non-planarity of the *∠*OCCH dihedral angle, are qualitatively recovered at the tight-binding level. Looking at the corresponding energy levels (Figure 5b), one finds further similarities between the two levels of theory, in that the *S*_0_/*S*_1_ MECP is located at around 4 eV, and therefore lower than the tristate structure at around 6 eV, relative to the ground state. Again, we must stress that obtaining accurate energetics with GFN0-xTB is outside the intended scope and parametrization of the method.^16^ However, our observations reveal that the method is well-suited for preoptimization and sampling of MECP geometries, even multistate MECPs, for subsequent optimization at a higher level of theory. Evidently, the effective one-electron approach based on the presented single-determinant treatments may require some manual adjustments, particularly for the optimization of the tristate MECP. Here, different orbital occupations that could represent the higher-lying excited states need to be tested. Given the computational efficiency of the approach, however, multiple combinations of orbital occupations can quickly be investigated. Optimization (or single-point evaluations) at a higher level of theory like CASCI or *hh*TDA-BHLYP-D3(BJ)/def2-SV(P) will then quickly reveal if the sampled MECPs correspond to relevant MECIs.

In conclusion, we have introduced a novel application of the non-self-consistent SQM method GFN0-xTB. The main results from the presented work are the following: (1) Given a specific orbital occupation, one can qualitatively describe higher-lying electronic states using only a single determinant composed of a single set of orthogonal orbitals. These orbitals are obtained via a single diagonalization of the non-self-consistent “Hückel-type” Hamiltonian and are used to describe different electronic states simultaneously. (2) Combining this method with a “derivative coupling vector-free” treatment enables the optimization of MECPs as estimates for excited-state MECIs. The approach is easily extended to tristate intersections. The computational efficiency of the non-self-consistent treatment allows for rapid testing of different reference occupations. (3) Calculated MECP geometries at the GFN0-xTB level are of similar quality to those obtained with GFN2-xTB for MECPs between the ground state and first excited states. In addition, the new scheme extends the capabilities of the previous approach^9^ to higher-state intersections. Overall, the structural features of the MECI are retained at a qualitative level, while larger deviations are expected for corresponding energetics. This result qualifies the presented scheme as suitable for application in metadynamics-based screening of MECPs^4,8,9,63^ and automated photoreaction analysis. Being parametrized for all elements up to radon (*Z* ≤ 86), GFN0-xTB is immediately applicable to a large variety of systems. Failures of GFN0-xTB are comparable to those previously observed for GFN2-xTB (e.g., missing pyramidalization features of the MECP in non-polar carbon-double bonds). Other known shortcomings, such as incorrect charge-transfer^12^ are also expected. (4) To the best of our knowledge, we here presented the first approach to describe non-Aufbau occupations via a modified Fermi-smearing technique to generate holes in the reference orbital occupation vector while maintaining robustness in molecular simulations.

As outlined above, single-reference SQM methods are the most simplified levels of theory at which a qualitatively correct description of the electronic structure is possible. Within the family of SQM methods, non-self-consistent variants such as GFN0-xTB provide the greatest computational cost savings. In the context of optimizing MECPs, this approach offers a speed-up of roughly 1–2 orders of magnitude compared to semiemipirical SCF variants. Thus, the outlined procedures may pave the way for developments of photochemistry-specific xTB-type Hamiltonians, e.g., by including Fock exchange,^48^ and automated workflows for photochemical applications.

## Supporting Information Available

The Supporting Information contains a zip archive with all optimized GFN0-xTB MECP geometries presented in the manuscript, including input files used to obtain these structures. Where appropriate, the respective *hh*TDA and FOMO-CASCI reference MECI geometries are also included. (ZIP)

## Notes

Open-source implementations of this publication can be found on GitHub at https://github.com/crest-lab/crest and https://github.com/pprcht/gfn0.

## References

[ref1] Feringa B. L. The Art of Building Small: From Molecular Switches to Motors (Nobel Lecture). Angew. Chem., Int. Ed. 2017, 56, 11060–11078. 10.1002/anie.201702979. 28851050

[ref2] Goulet-Hanssens A.; Eisenreich F.; Hecht S. Enlightening Materials with Photoswitches. Adv. Mater. 2020, 32, 1905966 10.1002/adma.201905966. 31975456

[ref3] Matsika S. Electronic Structure Methods for the Description of Nonadiabatic Effects and Conical Intersections. Chem. Rev. 2021, 121, 9407–9449. 10.1021/acs.chemrev.1c00074. 34156838

[ref4] Pieri E.; Lahana D.; Chang A. M.; Aldaz C. R.; Thompson K. C.; Martínez T. J. The non-adiabatic nanoreactor: towards the automated discovery of photochemistry. Chem. Sci. 2021, 12, 7294–7307. 10.1039/D1SC00775K. 34163820 PMC8171323

[ref5] Lykhin A. O.; Kaliakin D. S.; dePolo G. E.; Kuzubov A. A.; Varganov S. A. Nonadiabatic transition state theory: Application to intersystem crossings in the active sites of metal-sulfur proteins. Int. J. Quantum Chem. 2016, 116, 750–761. 10.1002/qua.25124.

[ref6] Plaza M.; Großkopf J.; Breitenlechner S.; Bannwarth C.; Bach T. Photochemical Deracemization of Primary Allene Amides by Triplet Energy Transfer: A Combined Synthetic and Theoretical Study. J. Am. Chem. Soc. 2021, 143, 11209–11217. 10.1021/jacs.1c05286. 34279085

[ref7] Inamori M.; Ikabata Y.; Yoshikawa T.; Nakai H. Unveiling controlling factors of the S0/S1 minimum energy conical intersection (2): Application to penalty function method. J. Chem. Phys. 2020, 152, 144108 10.1063/1.5142592. 32295362

[ref8] Lindner J. O.; Sultangaleeva K.; Röhr M. I. S.; Mitrić R. metaFALCON: A Program Package for Automatic Sampling of Conical Intersection Seams Using Multistate Metadynamics. J. Chem. Theory Comput. 2019, 15, 3450–3460. 10.1021/acs.jctc.9b00029. 30995044

[ref9] Pracht P.; Bannwarth C. Fast Screening of Minimum Energy Crossing Points with Semiempirical Tight-Binding Methods. J. Chem. Theory Comput. 2022, 18, 6370–6385. 10.1021/acs.jctc.2c00578. 36121838

[ref10] Pracht P.; Bohle F.; Grimme S. Automated exploration of the low-energy chemical space with fast quantum chemical methods. Phys. Chem. Chem. Phys. 2020, 22, 7169–7192. 10.1039/C9CP06869D. 32073075

[ref11] Bannwarth C.; Ehlert S.; Grimme S. GFN2-xTB – An Accurate and Broadly Parametrized Self-Consistent Tight-Binding Quantum Chemical Method with Multipole Electrostatics and Density-Dependent Dispersion Contributions. J. Chem. Theory Comput. 2019, 15, 1652–1671. 10.1021/acs.jctc.8b01176. 30741547

[ref12] Bannwarth C.; Caldeweyher E.; Ehlert S.; Hansen A.; Pracht P.; Seibert J.; Spicher S.; Grimme S. Extended tight-binding quantum chemistry methods. WIREs Comput. Mol. Sci. 2021, 11, e01493 10.1002/wcms.1493.

[ref13] Tuna D.; Sobolewski A. L.; Domcke W. Photochemical Mechanisms of Radiationless Deactivation Processes in Urocanic Acid. J. Phys. Chem. B 2014, 118, 976–985. 10.1021/jp411818j. 24397532

[ref14] Coe J. D.; Martínez T. J. Ab Initio Molecular Dynamics of Excited-State Intramolecular Proton Transfer around a Three-State Conical Intersection in Malonaldehyde. J. Phys. Chem. A 2006, 110, 618–630. 10.1021/jp0535339. 16405334

[ref15] Kunze L.; Hansen A.; Grimme S.; Mewes J.-M. PCM-ROKS for the Description of Charge-Transfer States in Solution: Singlet–Triplet Gaps with Chemical Accuracy from Open-Shell Kohn–Sham Reaction-Field Calculations. J. Phys. Chem. Lett. 2021, 12, 8470–8480. 10.1021/acs.jpclett.1c02299. 34449230

[ref16] Pracht P.; Caldeweyher E.; Ehlert S.; Grimme S. A Robust Non-Self-Consistent Tight-Binding Quantum Chemistry Method for large Molecules. ChemRxiv, 2019. 10.26434/chemrxiv.8326202.v1.

[ref17] Yarkony D. R. Marching along Ridges. Efficient Location of Energy-Minimized Conical Intersections of Two States Using Extrapolatable Functions. J. Phys. Chem. A 2004, 108, 3200–3205. 10.1021/jp0374354.

[ref18] Levine B. G.; Coe J. D.; Martínez T. J. Optimizing Conical Intersections without Derivative Coupling Vectors: Application to Multistate Multireference Second-Order Perturbation Theory (MS-CASPT2). J. Phys. Chem. B 2008, 112, 405–413. 10.1021/jp0761618. 18081339

[ref19] Maeda S.; Harabuchi Y.; Taketsugu T.; Morokuma K. Systematic Exploration of Minimum Energy Conical Intersection Structures near the Franck–Condon Region. J. Phys. Chem. A 2014, 118, 12050–12058. 10.1021/jp507698m. 25259835

[ref20] Aldaz C.; Kammeraad J. A.; Zimmerman P. M. Discovery of conical intersection mediated photochemistry with growing string methods. Phys. Chem. Chem. Phys. 2018, 20, 27394–27405. 10.1039/C8CP04703K. 30357173 PMC6532651

[ref21] Baek Y. S.; Lee S.; Filatov M.; Choi C. H. Optimization of Three State Conical Intersections by Adaptive Penalty Function Algorithm in Connection with the Mixed-Reference Spin-Flip Time-Dependent Density Functional Theory Method (MRSF-TDDFT). J. Phys. Chem. A 2021, 125, 1994–2006. 10.1021/acs.jpca.0c11294. 33651623

[ref22] Dicks L.; Wales D. J. Elucidating the solution structure of the K-means cost function using energy landscape theory. J. Chem. Phys. 2022, 156, 054109 10.1063/5.0078793. 35135278

[ref23] Grimme S. Exploration of Chemical Compound, Conformer, and Reaction Space with Meta-Dynamics Simulations Based on Tight-Binding Quantum Chemical Calculations. J. Chem. Theory Comput. 2019, 15, 2847–2862. 10.1021/acs.jctc.9b00143. 30943025

[ref24] Sutto L.; Marsili S.; Gervasio F. L. New advances in metadynamics. WIREs Comput. Mol. Sci. 2012, 2, 771–779. 10.1002/wcms.1103.

[ref25] Hartke B. Global optimization. WIREs Comput. Mol. Sci. 2011, 1, 879–887. 10.1002/wcms.70.

[ref26] Wales D. J. Exploring Energy Landscapes. Annu. Rev. Phys. Chem. 2018, 69, 401–425. 10.1146/annurev-physchem-050317-021219. 29677468

[ref27] Yarkony D. R. Nonadiabatic Quantum Chemistry – Past, Present, and Future. Chem. Rev. 2012, 112, 481–498. 10.1021/cr2001299. 22050109

[ref28] Nikiforov A.; Gamez J. A.; Thiel W.; Huix-Rotllant M.; Filatov M. Assessment of approximate computational methods for conical intersections and branching plane vectors in organic molecules. J. Chem. Phys. 2014, 141, 124122 10.1063/1.4896372. 25273427

[ref29] Spörkel L.; Cui G.; Thiel W. Photodynamics of Schiff Base Salicylideneaniline: Trajectory Surface-Hopping Simulations. J. Phys. Chem. A 2013, 117, 4574–4583. 10.1021/jp4028035. 23650926

[ref30] Niehaus T. A. Ground-to-excited derivative couplings for the density functional-based tight-binding method: semi-local and long-range corrected formulations. Theor. Chem. Acc. 2021, 140, 34 10.1007/s00214-021-02735-y.

[ref31] Niehaus T. A. Exact non-adiabatic coupling vectors for the time-dependent density functional based tight-binding method. J. Chem. Phys. 2023, 158, 054103 10.1063/5.0136838. 36754796

[ref32] Grimme S.; Bannwarth C.; Shushkov P. A Robust and Accurate Tight-Binding Quantum Chemical Method for Structures, Vibrational Frequencies, and Noncovalent Interactions of Large Molecular Systems Parametrized for All spd-Block Elements (*Z* = 1 – 86). J. Chem. Theory Comput. 2017, 13, 1989–2009. 10.1021/acs.jctc.7b00118. 28418654

[ref33] Kutta R. J.; Großkopf J.; van Staalduinen N.; Seitz A.; Pracht P.; Breitenlechner S.; Bannwarth C.; Nuernberger P.; Bach T. Multifaceted View on the Mechanism of a Photochemical Deracemization Reaction. J. Am. Chem. Soc. 2023, 145, 2354–2363. 10.1021/jacs.2c11265. 36660908

[ref34] Roothaan C. C. J. New Developments in Molecular Orbital Theory. Rev. Mod. Phys. 1951, 23, 69–89. 10.1103/RevModPhys.23.69.

[ref35] Hall G. G. The Molecular Orbital Theory of Chemical Valency. VIII. A Method of Calculating Ionization Potentials. Proc. R. Soc. London A 1951, 205, 541–552. 10.1098/rspa.1951.0048.

[ref36] Mermin N. D. Thermal Properties of the Inhomogeneous Electron Gas. Phys. Rev. 1965, 137, A1441–A1443. 10.1103/PhysRev.137.A1441.

[ref37] Cembran A.; Bernardi F.; Garavelli M.; Gagliardi L.; Orlandi G. On the Mechanism of the cis-trans Isomerization in the Lowest Electronic States of Azobenzene: S0, S1, and T1. J. Am. Chem. Soc. 2004, 126, 3234–3243. 10.1021/ja038327y. 15012153

[ref38] Yu J. K.; Bannwarth C.; Hohenstein E. G.; Martínez T. J. Ab Initio Nonadiabatic Molecular Dynamics with Hole–Hole Tamm–Dancoff Approximated Density Functional Theory. J. Chem. Theory Comput. 2020, 16, 5499–5511. 10.1021/acs.jctc.0c00644. 32786902

[ref39] Reimann M.; Teichmann E.; Hecht S.; Kaupp M. Solving the Azobenzene Entropy Puzzle: Direct Evidence for Multi-State Reactivity. J. Chem. Phys. Lett. 2022, 13, 10882–10888. 10.1021/acs.jpclett.2c02838. 36394331

[ref40] Bannwarth C.; Yu J. K.; Hohenstein E. G.; Martínez T. J. Hole–hole Tamm–Dancoff-approximated density functional theory: A highly efficient electronic structure method incorporating dynamic and static correlation. J. Chem. Phys. 2020, 153, 024110 10.1063/5.0003985. 32668944

[ref41] Seritan S.; Bannwarth C.; Fales B. S.; Hohenstein E. G.; Kokkila-Schumacher S. I. L.; Luehr N.; Snyder J. W.; Song C.; Titov A. V.; Ufimtsev I. S.; Martínez T. J. TeraChem: Accelerating electronic structure and ab initio molecular dynamics with graphical processing units. J. Chem. Phys. 2020, 152, 224110 10.1063/5.0007615. 32534542 PMC7928072

[ref42] Seritan S.; Bannwarth C.; Fales B. S.; Hohenstein E. G.; Isborn C. M.; Kokkila-Schumacher S. I. L.; Li X.; Liu F.; Luehr N.; Snyder J. W. Jr.; Song C.; Titov A. V.; Ufimtsev I. S.; Wang L.-P.; Martínez T. J. TeraChem: A graphical processing unit-accelerated electronic structure package for large-scale ab initio molecular dynamics. WIREs Comput. Mol. Sci. 2021, 11, e1494 10.1002/wcms.1494.

[ref43] Slavíček P.; Martínez T. J. Ab initio floating occupation molecular orbital-complete active space configuration interaction: An efficient approximation to CASSCF. J. Chem. Phys. 2010, 132, 234102 10.1063/1.3436501. 20572684

[ref44] Hollas D.; Šištík L.; Hohenstein E. G.; Martínez T. J.; Slavíček P. Nonadiabatic Ab Initio Molecular Dynamics with the Floating Occupation Molecular Orbital-Complete Active Space Configuration Interaction Method. J. Chem. Theory Comput. 2018, 14, 339–350. 10.1021/acs.jctc.7b00958. 29207238

[ref45] Granucci G.; Toniolo A. Molecular gradients for semiempirical CI wavefunctions with floating occupation molecular orbitals. Chem. Phys. Lett. 2000, 325, 79–85. 10.1016/S0009-2614(00)00691-6.

[ref46] Granucci G.; Persico M.; Toniolo A. Direct semiclassical simulation of photochemical processes with semiempirical wave functions. J. Chem. Phys. 2001, 114, 10608–10615. 10.1063/1.1376633.

[ref47] Coutsias E. A.; Seok C.; Dill K. A. Using quaternions to calculate RMSD. J. Comput. Chem. 2004, 25, 1849–1857. 10.1002/jcc.20110. 15376254

[ref48] Bannwarth C.; Martínez T. J. SQMBox: Interfacing a semiempirical integral library to modular ab initio electronic structure enables new semiempirical methods. J. Chem. Phys. 2023, 158, 074109 10.1063/5.0132776. 36813714

[ref49] Kratz T.; Steinbach P.; Breitenlechner S.; Storch G.; Bannwarth C.; Bach T. Photochemical Deracemization of Chiral Alkenes via Triplet Energy Transfer. J. Am. Chem. Soc. 2022, 144, 10133–10138. 10.1021/jacs.2c02511. 35658423

[ref50] Olivucci M.; Bernardi F.; Celani P.; Ragazos I.; Robb M. A. Excited-state cis-trans isomerization of cis-hexatriene. A CAS-SCF computational study. J. Am. Chem. Soc. 1994, 116, 1077–1085. 10.1021/ja00082a033.

[ref51] Schneider R.; Domcke W. S1-S2 Conical intersection and ultrafast S2 → S1 Internal conversion in pyrazine. Chem. Phys. Lett. 1988, 150, 235–242. 10.1016/0009-2614(88)80034-4.

[ref52] Lim J. S.; Kim S. K. Experimental probing of conical intersection dynamics in the photodissociation of thioanisole. Nat. Chem. 2010, 2, 627–632. 10.1038/nchem.702. 20651723

[ref53] Merchán M.; Serrano-Andrés L. Ultrafast Internal Conversion of Excited Cytosine via the Lowest *ππ*^*^ Electronic Singlet State. J. Am. Chem. Soc. 2003, 125, 8108–8109. 10.1021/ja0351600. 12837073

[ref54] Gómez I.; Reguero M.; Robb M. A. Efficient Photochemical Merocyanine-to-Spiropyran Ring Closure Mechanism through an Extended Conical Intersection Seam. A Model CASSCF/CASPT2 Study. J. Phys. Chem. A 2006, 110, 3986–3991. 10.1021/jp056208u. 16539421

[ref55] Credo Chung W.; Lan Z.; Ohtsuki Y.; Shimakura N.; Domcke W.; Fujimura Y. Conical intersections involving the dissociative 1*π′sigma*^*^ state in 9H-adenine: a quantum chemical ab initio study. Phys. Chem. Chem. Phys. 2007, 9, 2075–2084. 10.1039/B618745E. 17464388

[ref56] Fang Y.-G.; Valverde D.; Mai S.; Canuto S.; Borin A. C.; Cui G.; González L. Excited-State Properties and Relaxation Pathways of Selenium-Substituted Guanine Nucleobase in Aqueous Solution and DNA Duplex. J. Phys. Chem. B 2021, 125, 1778–1789. 10.1021/acs.jpcb.0c10855. 33570942 PMC8023715

[ref57] Valverde D.; Mai S.; Canuto S.; Borin A. C.; González L. Ultrafast Intersystem Crossing Dynamics of 6-Selenoguanine in Water. JACS Au 2022, 2, 1699–1711. 10.1021/jacsau.2c00250. 35911449 PMC9327080

[ref58] Kistler K. A.; Matsika S. Three-state conical intersections in cytosine and pyrimidinone bases. J. Chem. Phys. 2008, 128, 215102 10.1063/1.2932102. 18537450

[ref59] Coe J. D.; Martínez T. J. Competitive Decay at Two- and Three-State Conical Intersections in Excited-State Intramolecular Proton Transfer. J. Am. Chem. Soc. 2005, 127, 4560–4561. 10.1021/ja043093j. 15796506

[ref60] Fang W.-H.; Phillips D. L. The Crucial Role of the S1/T2/T1 Intersection in the Relaxation Dynamics of Aromatic Carbonyl Compounds upon *n* → π* Excitation. ChemPhysChem 2002, 3, 889–892. 10.1002/1439-7641(20021018)3:10<889::AID-CPHC889>3.0.CO;2-U.

[ref61] Matsika S. Radiationless Decay of Excited States of Uracil through Conical Intersections. J. Phys. Chem. A 2004, 108, 7584–7590. 10.1021/jp048284n.

[ref62] Matsika S. Three-State Conical Intersections in Nucleic Acid Bases. J. Phys. Chem.A 2005, 109, 7538–7545. 10.1021/jp0513622. 16834123

[ref63] Lindner J. O.; Röhr M. I. S.; Mitrić R. Multistate metadynamics for automatic exploration of conical intersections. Phys. Rev. A 2018, 97, 052502 10.1103/PhysRevA.97.052502.

